# Effect of Surface Ligands in Perovskite Nanocrystals: Extending in and Reaching out

**DOI:** 10.1021/acs.accounts.0c00712

**Published:** 2021-02-11

**Authors:** Miri Kazes, Thumu Udayabhaskararao, Swayandipta Dey, Dan Oron

**Affiliations:** Department of Molecular Chemistry and Materials Science, Weizmann Institute of Science, Rehovot 7610001, Israel

## Abstract

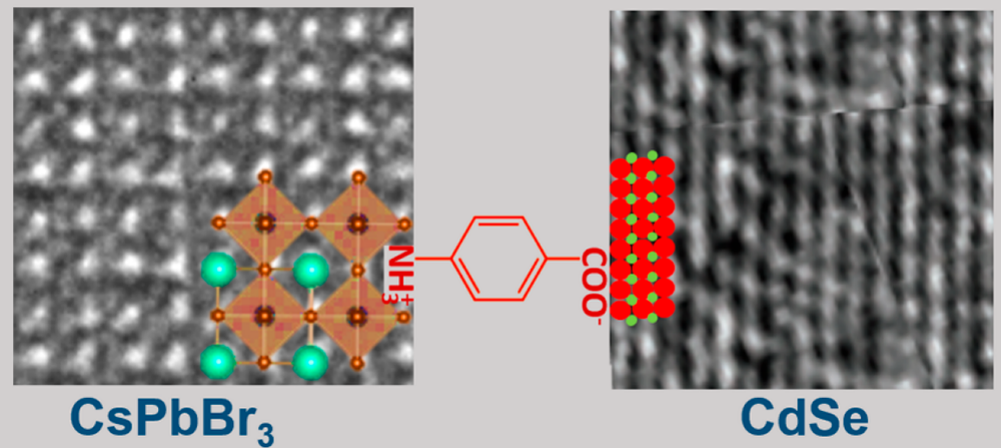

The rediscovery of the halide perovskite class of compounds and, in particular, the organic and inorganic lead halide perovskite (LHP) materials and lead-free derivatives has reached remarkable landmarks in numerous applications. First among these is the field of photovoltaics, which is at the core of today’s environmental sustainability efforts. Indeed, these efforts have born fruit, reaching to date a remarkable power conversion efficiency of 25.2% for a double-cation Cs, FA lead halide thin film device. Other applications include light and particle detectors as well as lighting. However, chemical and thermal degradation issues prevent perovskite-based devices and particularly photovoltaic modules from reaching the market. The soft ionic nature of LHPs makes these materials susceptible to delicate changes in the chemical environment. Therefore, control over their interface properties plays a critical role in maintaining their stability. Here we focus on LHP nanocrystals, where surface termination by ligands determines not only the stability of the material but also the crystallographic phase and crystal habit. A surface analysis of nanocrystal interfaces revealed the involvement of Brønsted type acid–base equilibrium in the modification of the ligand moieties present, which in turn can invoke dissolution and recrystallization into the more favorable phase in terms of minimization of the surface energy. A large library of surface ligands has already been developed showing both good chemical stability and good electronic surface passivation, resulting in near-unity emission quantum yields for some materials, particularly CsPbBr_3_. However, most of those ligands have a large organic tail hampering charge carrier transport and extraction in nanocrystal-based solid films.

The unique perovskite structure that allows ligand substitution in the surface A (cation) sites and the soft ionic nature is expected to allow the accommodation of large dipoles across the perovskite crystal. This was shown to facilitate electron transfer across a molecular linked single-particle junction, creating a large built-in field across the junction nanodomains. This strategy could be useful for implementing LHP NCs in a p–n junction photovoltaic configuration as well as for a variety of electronic devices. A better understanding of the surface propeties of LHP nanocrystals will also enable better control of their growth on surfaces and in confined volumes, such as those afforded by metal–organic frameworks, zeolites, or chemically patterened surfaces such as anodic alumina, which have already been shown to significantly alter the properties of in-situ-grown LHP materials.

## Key References

- UdayabhaskararaoT.; KazesM.; HoubenL.; LinH.; OronD.Nucleation, Growth, and Structural Transformations of Perovskite Nanocrystals. Chem. Mater.2017, 29( (3), ), 1302–130810.1021/acs.chemmater.6b04841.^1^*Pointing to the common attributes of previously reported lead halide NC syntheses. Shows that the synthesis mechanism goes through two stages: seed mediated nucleation and growth by self-assembly*.
- UdayabhaskararaoT.; HoubenL.; CohenH.; MenahemM.; PinkasI.; AvramL.; WolfT.; TeitelboimA.; LeskesM.; YaffeO.; OronD.; KazesM.. A Mechanistic Study of Phase Transformation in Perovskite Nanocrystals Driven by Ligand Passivation. Chem. Mater.2018, 30( (1), ), 84–9310.1021/acs.chemmater.7b02425.^2^*Shows the eminent role of the nature of surface ligands in perovskite NC phase transformations and clarifies the structural mechanism of crystallographic phase change from 0D Cs*_*4*_*PbX*_*6*_*to 3D CsPbX*_*3*_*perovskite*.
- DeyS.; CohenH.; PinkasI.; LinH.; KazesM.; OronD.Band Alignment and Charge Transfer in CsPbBr_3_-CdSe Nanoplatelet Hybrids Coupled by Molecular Linkers. J. Chem. Phys.2019, 151( (17), ), 17470410.1063/1.512455231703516.^3^*First example and characterization of a p–n-like junction of a perovskite-CdSe NCs hybrid*.

## 1 Introduction

Lead-halide perovskite (LHP) film-based optoelectronic applications such as solar cells, radiation detectors, and LEDs have reached impressive performance in the past decade. Still, there are significant technological challenges in the production of thin-film-based devices, encouraging the development of advanced protocols for colloidal perovskite NC synthesis. In turn, knowledge gathered from colloidal NC research, down to the atomic scale, is being implemented in improved methods for thin-film-based devices.^4^ A key component of colloidal nanocrystalline materials is the surface ligands. Surface ligands, in particular, in smaller NCs, owing to the large surface-to-volume ratio, have great importance in many aspects that are of utmost importance to the control and optimization of optoelectronic device design and performance. During synthesis, surface ligands serve as a major control knob in determining the size, shape, and crystalline habit of the NCs. Surface ligands have a dramatic effect on optical properties such as the PL QY, Stokes shift, and chirality. To a significant extent, they also govern the characteristics of interfaces between LHPs and other materials, enabling the control of charge carrier transport properties and even electronic band alignment via control of the molecular dipoles.^5−7^

In LHPs, the surface/bulk interface is much more subtle than in typical covalent semiconductor NCs owing to their soft ionic bonding nature and unique cagelike structure that favor surface substitution and reconstruction over adsorption.^8^ The 3D crystal structure of perovskite, described by the chemical formula ABX_3_, is constructed from corner-sharing lead halide [BX_6_]^4–^ octahedra with an organic or inorganic A-site cation (e.g., MA^+^, FA^+^, Cs^+^) occupying the central void between the octahedra. At the surface, where the atomic periodicity is interrupted, the crystal surface can terminate with either metal halide units or A-site cations. Because LHPs possess particularly weak ionic bonding character, their surface terminations are highly dependent on their synthesis and/or processing history. Moreover, the soft ionic nature of LHPs renders them liable to crystal distortions where surface effects can extend well into the bulk of the crystal.^9−11^

When A sites are occupied by groups that are too large, such as the long-chain alkyl aminmmonium cations, LHPs turn into the 2D layered structure (i.e., Ruddlesden–Popper perovskites)^12^ to form quantum well superlattices, thus providing an additional degree of freedom to tune the intrinsic physical features, including the optical band gap, exciton binding energy, and dielectric constant.^13,14^

In this Account, we highlight the role of molecular surface ligands in colloidal LHP nanocrystals, beginning with a discussion of their role in synthesis and crystal phase control, followed by their effect on the electronic properties at heterojunctions between LHPs and other semiconductors through the example of a CsPbBr_3_–CdSe hybrid structure.

## 2 Role of Molecular Surface Ligands in Colloidal Perovskite NC Synthesis

Two main routes for the synthesis of LHP NCs have been demonstrated to date: ligand-assisted reprecipitation and the hot-injection method. In ligand-assisted reprecipitation, the inorganic salt precursors and organic amine and carboxylic acid ligands are dissolved in a polar solvent such as DMF acting as a good solvent, while a nonpolar solvent such as toluene acts as a poor solvent to promote the reprecipitation process. In this case, shape control was demonstrated depending on the aliphatic chain length of the ligands used.^15^ A second route, pioneered by Schmidt et al.,^16^ applied the hot-injection method strategy typically used for semiconductor and metal QDs for the growth of hybrid organic–inorganic MAPbBr_3_ NCs.^16^ The synthesis involved the reaction of a metal salt in the presence of oleic acid (OA) in octadecene (ODE) with the injection of the ammonium cation at a moderate temperature of 80 °C. This synthesis method relied on the addition of a longer ammonium cation that cannot be incorporated into the perovskite crystal structure, thus arresting the crystal growth, leading to NC formation.

Both synthesis methodologies pointed to the importance of acid–base reactions controlling the formation of ammonium ions in the reaction mixture. For example, thickness control of MAPbBr_3_ nanoplatelets (NPLs) was achieved by tuning the acidity of the reaction mixture.^17,18^ The effect of pH was explained by the formation of ammonium through an amine protonation by an acid, resulting in preferential binding, where the ammonium competes with Cs^+^ ions on the surface of the growing platelets and selectively slows the growth along the vertical direction, promoting anisotropic growth.^19−21^ Further work revealed the dependence of LHP NC morphologies on the acidic and basic hydrocarbons, such as amines and carboxylic acids, and their ratios and chain lengths.^22−24^ The use of shorter-chain amines leads to thinner NPLs, the use of shorter-chain carboxylic acids leads to larger NCs,^23^ and the use of only OA produces orthorhombic nanocubes with no shape control.^25^ There is a prominent effect on the end product and surface chemistry of LHP synthesis under specific synthesis conditions such as temperature, solvent polarity, and ligand-to-metal molar ratios which in turn can affect different equilibrium pathways such as PbX_2_ dissociation, metal–ligand complexation, and, of course, acid–base reactions.^21^ In fact, a careful investigation of all reported LHP NP syntheses to date seems to show that all reactions actually follow the same general main attributes. First, the involvement of ammonium species in surface passivation was shown by De Roo et al.^26^ Second, the ammonium species can be formed by amine protonation that can be realized either by direct reaction with an organic acid at high temperatures or by the addition of polar solvents, even at room temperature. Third, there is preferential binding of ammonium over the carboxylate ligand to the perovskite surface, replacing the A-site cation and thus affecting the growth kinetics and the obtained size and shape.

### 2.1 Effect of Polar Molecular Agents in the Perovskite Nucleation Reaction

**Figure 1:**
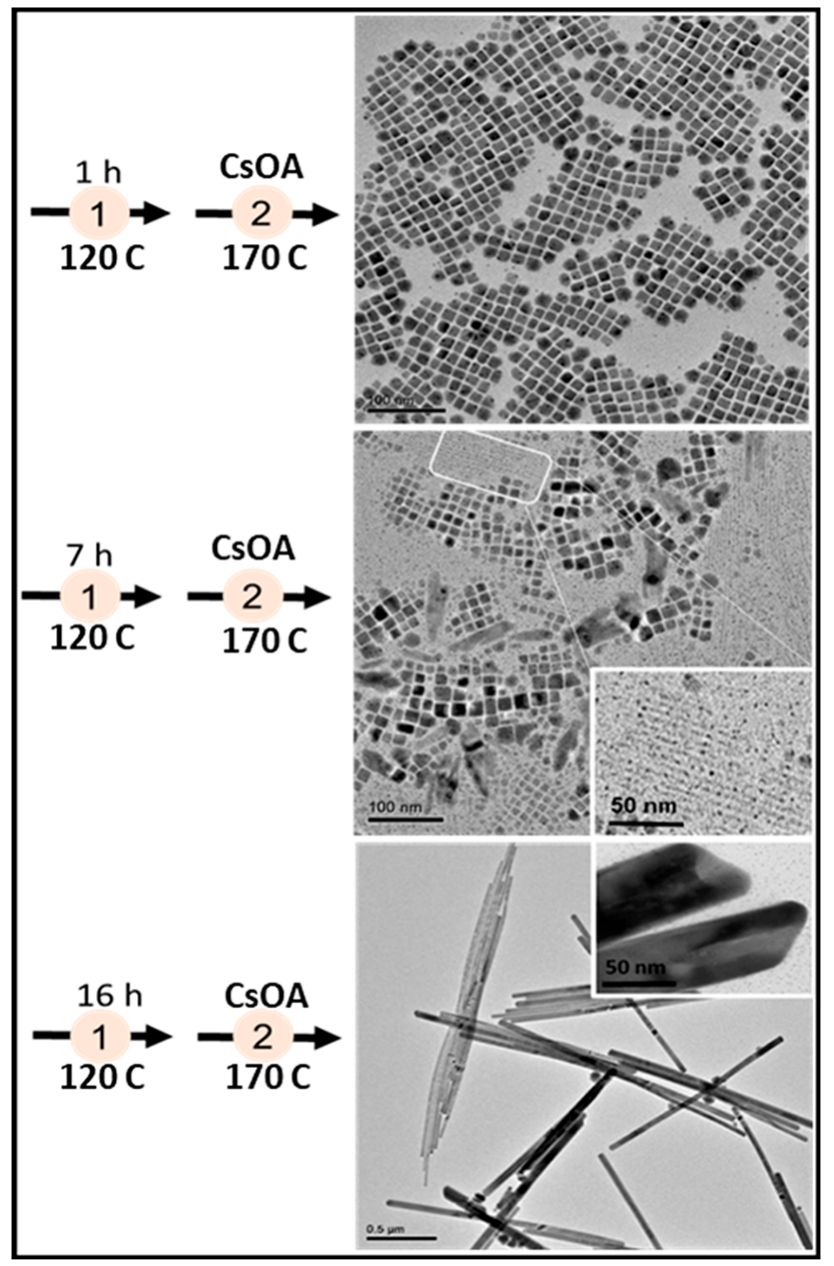
Effect of seed aging time (i.e., step 1) on the morphology of intermediate CsPbI_3_ (i.e., step 2). As the aging time is increased, the reaction product of stage 1 change from cubes for 1 h of aging time to mixed cubes and thin wires for 7 h of aging and long wires and tubes for 16 h of aging time.

The synthesis of colloidal LHP NCs, pioneered by Protesescu et al., uses PbBr_2_ complexed by OA and OLAm in a noncoordinating ODE medium and CsOA solution injected at an elevated temperature followed by fast cooling.^27^ Interestingly, different end products can be obtained even without any apparent change in the composition of the reactants. As it turns out (and quite out of the ordinary situation in “typical” colloidal semiconductor nanocrystal synthesis, such as in the case of CdSe), the reaction is so sensitive that even a variation of the reaction time of the PbI_2_ precursor with OA and OLAm prior to the addition of CsOA precursor can significantly affect the end product. We tried to elucidate the origin of this phenomenon by relating it either to the different nucleation kinetics or to a chemical reaction of the ligands.^1^ First, we have found that Pb^0^ NPs are formed during the aging time, the remnants of which are still embedded in the end product. This suggests that Pb^0^ NPs serve as seeds for LHP NP nucleation. Second, while cube-shaped CsPbI_3_ NCs were obtained after 1 h of aging time, micrometer-sized NWs were obtained after 16 h of aging time, along with a mixture of both in between (Figure 1). The major factor controlling the end product was neither the seed size nor the concentration but rather the polar molecular species forming during the aging process. This conclusion was further supported by the single-step formation of NPLs and nanowires upon injection of small amounts of polar additives into the reaction flask. An extension to this idea was recently reported by Chakrabarty et al., where cesium cholate in a small volume of methanol was used as the Cs precursor to synthesize differently shaped LHPs by controlling the polarity of the reaction mixture.^28^ Further support for this mechanism is provided by the work of Grisorio et al., where two-dimensional [(RNH_3_)_2_(PbBr_4_)]*_n_* structures were shown to form during synthesis prior to the addition of the Cs precursor.^29^ De Roo et al. showed that CsPbBr_3_ NCs can be terminated either by oleylammonium bromide or oleylammonium carboxylate that can bind either to the metal or to the cation, and both are necessary to stabilize the CsPbX_3_ NCs in solution.^26^ Furthermore, there is a dynamic stabilization of oleylammonium bromide in solution, and the binding strength indeed relies on controlled acid–base equilibrium afforded by the presence of OA.^30^

### 2.2 Growth by Oriented Attachment Mediated by Molecular Polar Agents

**Figure 2:**
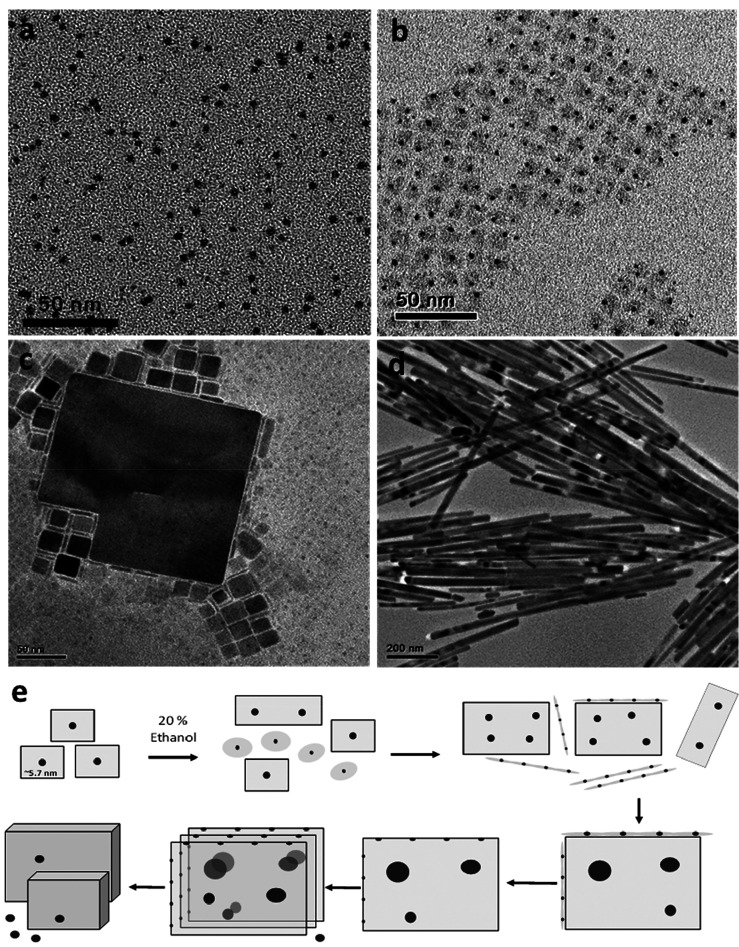
TEM image of the initial Pb^0^ NPs (a) serving as nucleation seeds for the synthesis of 5 nm CsPbBr_3_ NPLs (b). (c) Self-assembly of NPLs in the reaction mixture into larger cubes in the case of CsPbBr_3_ and wires for CsPbI_3_ (d). (e) Schematic representation of the conversion of nanocubes to bulk-type crystals through orientated attachment self-assembly.

Growth by oriented attachment into larger self-assembled structures can proceed either by the intentional destabilization of surface ligand passivation or by spontaneous gradual aggregation.^18,22,31^Figure 2 shows the oriented attachment of CsPbX_3_ NCs observed in the reaction mixture left to anneal over some time. Postsynthesis treatments which affect the surface composition of the NPs showed similar results. Bekenstein et al.^22^ showed that postsynthesis addition of alkenehalides to the reaction mixture at an elevated temperature of 110 °C produces only 2D thin sheets. In this case, the alkenehalide serves as a polar destabilizer, likely through complexation with OLAm. Pan et al. showed that purification with acetone removes the ammonium ligands but retains the carboxylates.^23^ These observations suggest that sufficient removal of the ammonium ligand plays a major role in the oriented attachment growth mode into 2D structures, while oleate ligands are retained on the surface. Growth into 3D structures is, in turn, promoted by the removal of both ammonium and oleate ligands.

## 3 Phase Transformations

The phase diagram of cesium lead halides enables the stable growth of materials with several ratios of CsX and PbX_2_ (where X is a halide).^32^ A 1:1 ratio yields the most commonly studied form, CsPbX_3_, featuring a three-dimensional (3D) network of corner-shared lead halide octahedra. A 1:2 ratio yields CsPb_2_X_5_, a two-dimensional (2D) layered perovskite derivate obtained from the three-dimensional (3D) analogue by slicing along crystallographic planes and the insertion of PbX_2_ planes. Finally, a 4:1 ratio yields Cs_4_PbX_6_, a quasi-zero-dimensional (0D) perovskite derivate with a recurring motif of isolated lead halide octahedra. Cs_4_PbX_6_, exhibiting highly localized optical excitations and a band gap in the near-ultraviolet (near-UV) region, is often also a recurrent byproduct of CsPbX_3_ syntheses.^33−35^ Intriguingly, a reversible transformation from CsPbX_3_ to Cs_4_PbX_6_ NCs can be induced by various methods. These include the addition of lead salts^36^ and the addition of thiols serving as a strong complexing agent for Pb^37^ via a two-phase polar/nonpolar reaction with water by extraction of CsBr into the water phase^38^ or by Cs extraction with a chelating agent.^39^ Other variants on these have also been reported in the litaerature.^40−42^ However, in all of these examples it is clear that either addition or extraction of one of the metal ions is responsible for the transformation.

We have shown that a similar transformation can be reversibly induced by much more subtle modifications to the LHP NP surface via ligand control, focusing on the mechanistics of phase transformation in terms of crystal structure and habit.

### 3.1 Ligand-Mediated Phase and Habit Transformations of Perovskite Nanocrystals

**Figure 3:**
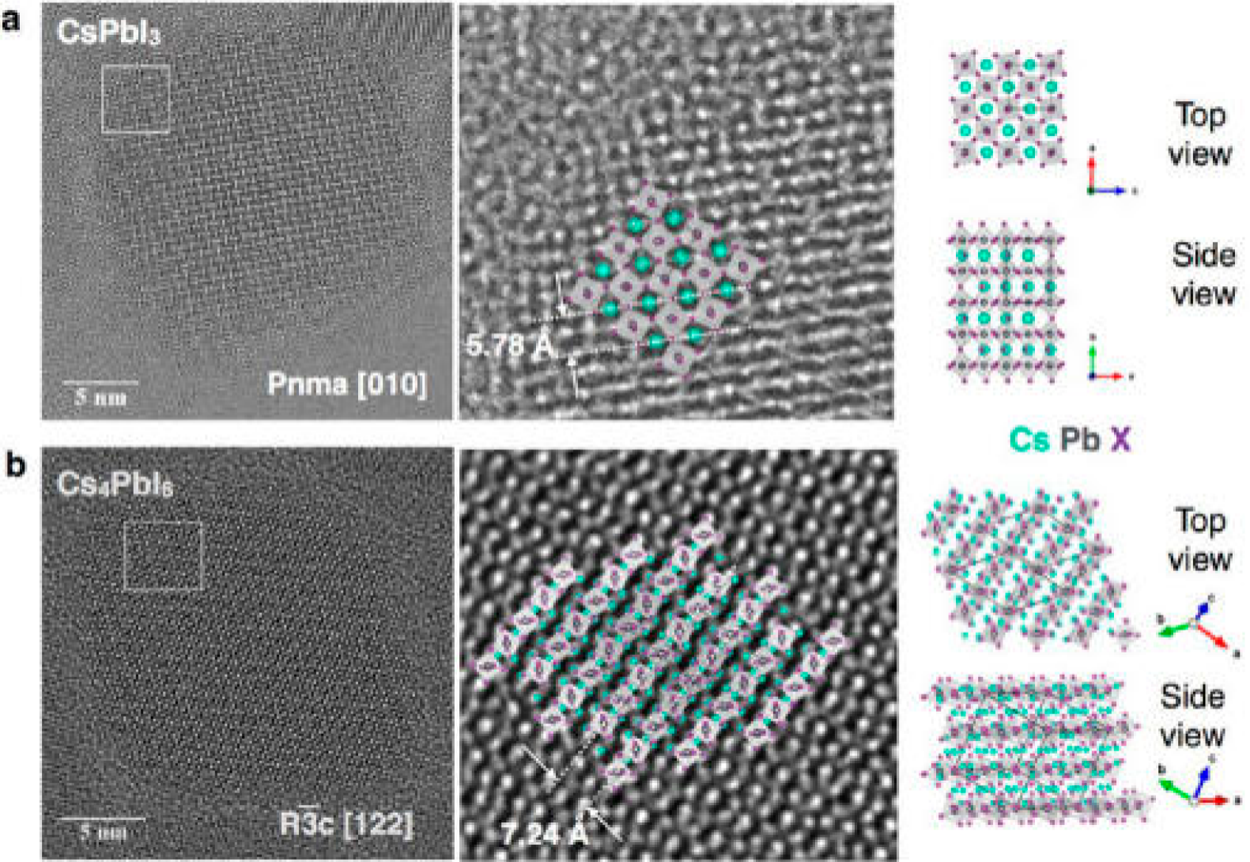
Atomic-resolution images of (a) CsPbI_3_ and (b) Cs_4_PbI_6_. CsPbI_3_ crystallizes in a perovskite crystal structure with orthorhombic distortion in which PbX_6_ octahedra are corner-sharing. The cubic crystals are bound by facets on (001) and (100) planes. The Cs_4_PbI_6_ structure is rhombohedral with space group *R*3*c*. The typical 2-fold symmetry of the high-resolution images of Cs_4_PbX_6_ is produced by the projection of chains of PbX_6_ octahedra in the [122] viewing direction. The habit is such that the rhombohedral crystals are formed by a layering of densely packed PbX_6_ with interlayers of cations.

Here we show how a robust reversible transformation from CsPbX_3_ to rhombohedral Cs_4_PbX_6_ can be achieved via control of the OA to OLAm Brønsted acid–base-type equilibrium. A small excess of OLAm over OA in a hexane solution of purified CsPbX_3_ NCs leads to the spontaneous transformation from orthorhombic CsPbX_3_ NCs to Cs_4_PbX_6_ NCs and vice versa even at room temperature. Evidence of the conversion is the change in luminescence color. The route for the structural transformation between CsPbI_3_ and Cs_4_PbI_6_ is illustrated in Figure 3, where TEM images show that during the conversion the cubic morphology of CsPbI_3_ NCs changes to rhombohedral.

The crystal structure of the cubic particles (Figure 3a) exhibits a characteristic tilt of the lead halide octahedra and is in agreement with orthorhombic space group *Pnma*. The cube facets of the CsPbI_3_ NCs are the trivial principal lattice planes (i.e., (100) and (001)). The rhombohedral Cs_4_PbX_6_ phase (Figure 3b) corresponds to the *R*3*c* crystal structure. All rhombohedra share a common [122] viewing direction with 2- fold symmetry. The rhomboidal cross section of the Cs_4_PbX_6_ crystals is determined by their (2–32) and (−2–12) side facets, while (012) planes represent the top and bottom facets. In this crystal habit, the rhombohedral particles are characterized by a stack of layers of densely packed PbX_6_ octahedra, alternating with an interlayer of Cs cations. The truncation of rhombohedra corners is found frequently and occurs on densely packed PbX_6_ octahedra as well. Therefore, the reversible transformation between the two phases involves a transition between a 3D network of corner-shared PbX_6_ octahedra in the orthorhombic phase to a 0D layered network of isolated PbX_6_ octahedra in the rhombohedral phase that are separated by cation planes. In relation to the NC surface, the orthorhombic CsPbX_3_ phase as seen in Figure 3a could have either a highly negatively or a highly positively charged surface (depending on the termination), while the facets of the rhombohedral Cs_4_PbX_6_ phase exhibit both PbX_6_ octahedra and Cs atoms and are thus expected to be closer to neutral.

Elucidating the transformation process requires combining data from several surface-sensitive spectroscopy modalities such as ^1^H NMR, FTIR, and XPS. Although each of these methods alone reveals only a partial description of the system, the combination gives a rather conclusive description of the major differences between CsPbBr_3_ and Cs_4_PbBr_6_. The ligand shell of CsPbBr_3_ is composed of bonded ammonium ligands substituting for the Cs atom vacancies on the partial Cs-terminating surface. In addition, Chen et al. showed that the oleate ligands bind to exposed Cs or Pb surface atoms.^43^^1^H NMR measurements suggests that the ligand shell of Cs_4_PbBr_6_ is likely composed of both OLAm and OA in a bonded state. In addition, Cs_4_PbBr_6_ surface ligands seem to have a more restricted spatial configuration possibly because of the rigidity of ammonium–oleate ion pair capping. This picture is in accordance with the Cs_4_PbBr_6_ surface composed of negatively charged PbBr_6_ octahedra that are partially balanced by in-plane Cs ions, thus requiring passivation by both negatively and positively charged ligands adsorbed on the surface. In contrast, for CsPbBr_3_, the highly ionic nature of the surface requires a strong ionic stabilization by ammonium substitution of Cs vacancies. Interestingly, when secondary aliphatic amines such as didodecylamine are used, such a phase transformation from CsPbBr_3_ to Cs_4_PbBr_6_ does not occur.^25^ An NMR study indicates that in this case both the acid and the secondary amine interact with the NC surface. However, a majority of the ligands present belong to oleate species by substitution of Br^–^ vacancies on a Pb-rich surface, while the secondary amines are believed to detach from the surface due to large steric repulsions between the ammonium groups.

The transformation mechanism we proposed involves a change in the surface ligand environment,^26,44^ followed by recrystallization induced by micelle formation^15^ or soft ligand templating.^22,45^ This mechanism is supported by the observation of intermediate stages in TEM images showing the gradual decomposition of cubic CsPbX_3_ NCs into lamellar structures, thin sheets, platelets, and amorphous material coexisting in solution, as also supported by the absorption spectra.

**Figure 4:**
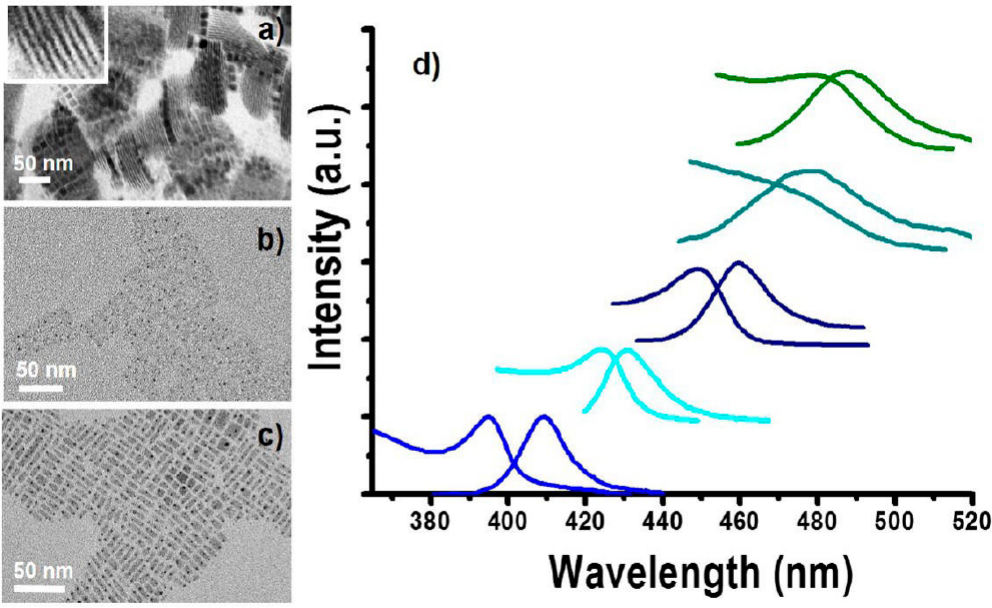
Selective transformation of Cs_4_PbBr_6_ NCs to CsPbBr_3_ NCs of different thicknesses. (a–c) TEM images of the CsPbBr_3_ samples emitting at 410, 432, and 490 nm, respectively. (d) Absorption and emission spectra of CsPbBr_3_ NCs. Five different absorption and emission peaks correspond to five different thicknesses (1–10 unit cells). Emission peaks from left to right: 410 nm (1 ML), 432 nm (2 MLs), 460 nm (5 MLs), 479 nm (8 MLs), and 488 nm (10 MLs).

For the backward transformation reaction from Cs_4_PbBr_6_ to CsPbBr_3_, by monitoring the evolution of the emission spectrum over the course of the reaction, we found that different peaks emerged and disappeared at discrete wavelengths corresponding to discrete thicknesses of several perovskite MLs (Figure 4). This suggests an exfoliation^46^ process aided by excess oleic acid followed by ionic sphere rearrangement and recrystallization as also suggested by Liu et al.^37^ This model is supported by Baranov et al.,^47^ where the analysis of the volume change of single Cs_4_PbBr_6_ NCs to CsPbBr_3_ gave rise to the conclusion that dissolution–recrystallization processes should indeed play an important role in this transformation and not just the gradual removal of CsBr from each individual NC.

### 3.2 Surface Thermodynamics

Bulk semiconductors tend to crystallize into a thermodynamically stable structure under ambient conditions, and metastable structures can be obtained only at elevated temperatures/pressures. In contrast, nanosized semiconductor colloidal crystallites with various crystal structures can be obtained under mild conditions. It is contemplated that metal–organic complex precursors of high reactivity are likely to rapidly decompose into a thermodynamically favored phase, while less reactive complexes lead to the formation of the kinetically (thermally) favored one. Moreover, it was shown that phase change can occur, controlled by the type of surface ligands.^48^ For example, the transformation of wurtzite CdSe into zinc blende and vice versa depends on carboxylic acid to amine surface passivation.^49,50^ Two main considerations should be taken into account in the case of perovskite NCs. The first is that in the more common II–VI or III–V semiconductor NCs the breaking of covalent bonds requires higher temperatures, with the highly ionic character of the metal halide perovskites rendering them much more liable for decomposition. Second, charge balance is required.

De Roo et al. show that there is a dynamic stabilization of oleylammonium bromide in solution and that the binding strength relies on controlled acid–base equilibrium.^26^ Quarta et al.^51^ reported a comprehensive study over a range of amine ligands (and their conjugated acids) with different basicity, chain length, and steric hindrance, demonstrating that the ligand binding affinity and the ligand-to-NC molar ratio (also through acid–base equilibrium) control the surface coordination.

In more detail, CsPbX_3_ can be viewed as constituted by a stoichiometric CsPbX_3_ core, which exposes an inner PbX_2_ shell and an outer, labile shell made up of −NH^3+^Br^–^ or Cs^+^–COO^–^ species. In addition, the binding of ligands at the surface can follow either a substitution mechanism or an addition mechanism such that −NH^3+^ replaces Cs^+^ on the surface and −COO^–^ replaces Br^–^ or that −NH^3+^ adsorbs to Br^–^ and −COO^–^ absorbs to Cs^+^.^51^ In addition, charge-neutral ligands (−NH_2_, chelating ligands) can induce the removal of PbX_2_, and positively charged species compete with Cs^+^ ions, by analogy to etching of metal chalcogenide NCs induced by amines. Namely, there is a trade-off between ligand binding on the surface and surface atoms being removed to expose the “inner” bulk atoms, which is intrinsically related to the solubility of the involved species. The binding energy of a ligand at the surface is governed by two parameters: the energy gain of formation of hydrogen bonds and the penalty of the surface reconstruction energy.

Specific synthesis conditions, such as ligand-to-metal molar ratios and temperature^21,51^ that affect the equilibrium of different reactions involved in the synthesis via acid–base reactions and PbX_2_ dissociation, have great cooperative effects on surface passivation, and there are many examples of ammonium–oleate^52^ and oleate-only^53^ ligand passivation motifs. However, global favored surface passivation seems to apply here: Ravi et al.^29^ confirmed the CsPbX_3_ surface passivation by OLA^+^ by DFT calculations, showing that the energy cost of the substitution mechanism for surface reconstruction is in fact low. In contrast, there is an energy gain by the formation of three hydrogen bonds among the −NH^3+^ moiety of oleylammonium and surrounding Br^–^ on the surface. This is corroborated by the experimental work of Smock et al.,^30^ where temperature-dependent ^1^H NMR spectroscopy was used to determine the thermodynamic exchange parameters of OA and OLA with the surface, concluding that although both ligand-exchange processes are indeed favorable at room temperature (negative Δ*G*s), for the exchange reaction with carboxylic acid, the enthalpy and entropy terms are positive in sign, while they are negative for OLA (energy gain). Most interestingly, OA/OLA-capped CsPbBr_3–*x*_I_*x*_ exchanged with oleylammonium iodide and lead undecenoate resulted in only low-density coverage of oleylammonium bromide on the surface and rapid morphological degradation as the iodide ratio increased, in contrast to similar CsPbBr_3_ NCs tested.^52^ This again indicates a scenario of favorable ligand affinity of ammonium over oleate to stabilize CsPbI_3_ in its optically active perovskite phase.^54^

Another example of the effect of surface thermodynamics is the use of alkyl phosphonic acids that produces an orthorhombic truncated octahedron through the favorable binding affinity to the Pb cation over the Cs one, thus exposing facets that are not observed in the orthorhombic phase produced by the typical acid–base type of reactions discussed above.^55^ A preferred substitution mechanism over adsorption is in stark contrast to the usual adsorption of organic ligands on the surface of typical covalent NCs. Moreover, this implies a unique interaction between the organic capping ligands and the inorganic core that can result in more extensive electronic interactions at the interface than in typical NCs.

## 4 Molecular Control of Perovskite-Based Junctions

A p–n junction structure is the common choice of electronic architecture in optoelectronic devices such as LEDs, photodiodes, and photovoltaics. A built-in electric field formed across the junction enhances the spatial separation and selective transport of the photoinduced electrons/holes, thus decreasing possible carrier recombination losses. In this respect, halide perovskites possess some unique properties as detailed below.

Efficient chemical doping was demonstrated by a MoO_3_ layer deposited on top of CH_3_NH_3_PbI_3_ nanosheets, showing a remarkably broad depletion region of up to 10 μm and a relatively high electric field of ∼0.5 eV; defect-induced self-doping as demonstrated by a MAPbI_3_ homojunction, created by different ratios of MA to Pb of two MAPbI_3_ layers grown one on top of the other;^56^ and a localized phase transition demonstrated by localized heating of a single CsSnI_3_ perovskite nanowire (NW) forming a p–n junction owing to the difference in formation energies of the cation and anion vacancies between the two phases.^57^ Band gap engineering through modification of the perovskite composition was demonstrated by anion exchange suggested for display pixel application.^58^ A gradient heterostructure photovoltaic cell based on a tolerance factor was demonstrated through the spontaneous doping of two sides of a FA_0.9_Cs_0.1_PbI_3_ perovskite thin film by “intolerant” n-type heteroatoms (Sb^3+^, In^3+^) with mismatched cation sizes and charge states.^59^ Band gap engineering was also demonstrated by the solution growth of vertically stacked double heterostructures and complex multilayer heterostructures of 2D lead iodide perovskites [(C_6_H_5_(CH_2_)_2_NH^3+^)_2_(MA)_*n*−1_PbnI_3*n*+1_]. Here the surface-bound ligands serve as spatial barriers to prevent ion migration across the junctions shown to enhance LED performance.^60,61^

However, although molecular linkers have been known to modify p–n junctions, they have not been explored in the field of perovskite structures.^62,63^ To explore the effect of molecular linkers on the electronic coupling in junction geometry, we chose to work with two prototypical systems, representing an LHP and a standard semiconductor coupled by an organic molecule. This system was also explored by Brumberg et al. but rather on a mixture of LHP and CdSe NCs with the native long-chain ligands, showing that the rate of electron transfer is dependent on the NC dimensionality.^64^ Here, NPs of CsPbBr_3_ and CdSe NPLs were linked by a bifunctionalized molecular linker, creating an analogue of nano p–n junctions with the built-in field depending on the nature of the linker molecule at the interface. Understanding the physics of charge transport across the molecular linkers as well as the characterization of interfacial electric fields and electrostatic changes taking place at related domains can provide a rational approach toward device design and optimization.

### 4.1 Charge-Transfer Processes in CsPbBr_3_/CdSe NPLs Hybrids Coupled by Molecular Linkers

The nature of electronic coupling and the effect of surface modifications on the interfacial charge-transfer dynamics upon hybrid formation were investigated for a system of lead halide-based perovskite NCs (CsPbBr_3_) and 2D colloidal II–VI semiconductor NCs (CdSe NPLs)—cross-linked *para*-aminobenzoic acid (PABA) and glycine (Gly) molecular ligands—and compared with a mixture of the two species with their native long-chain ligands.

**Figure 5:**
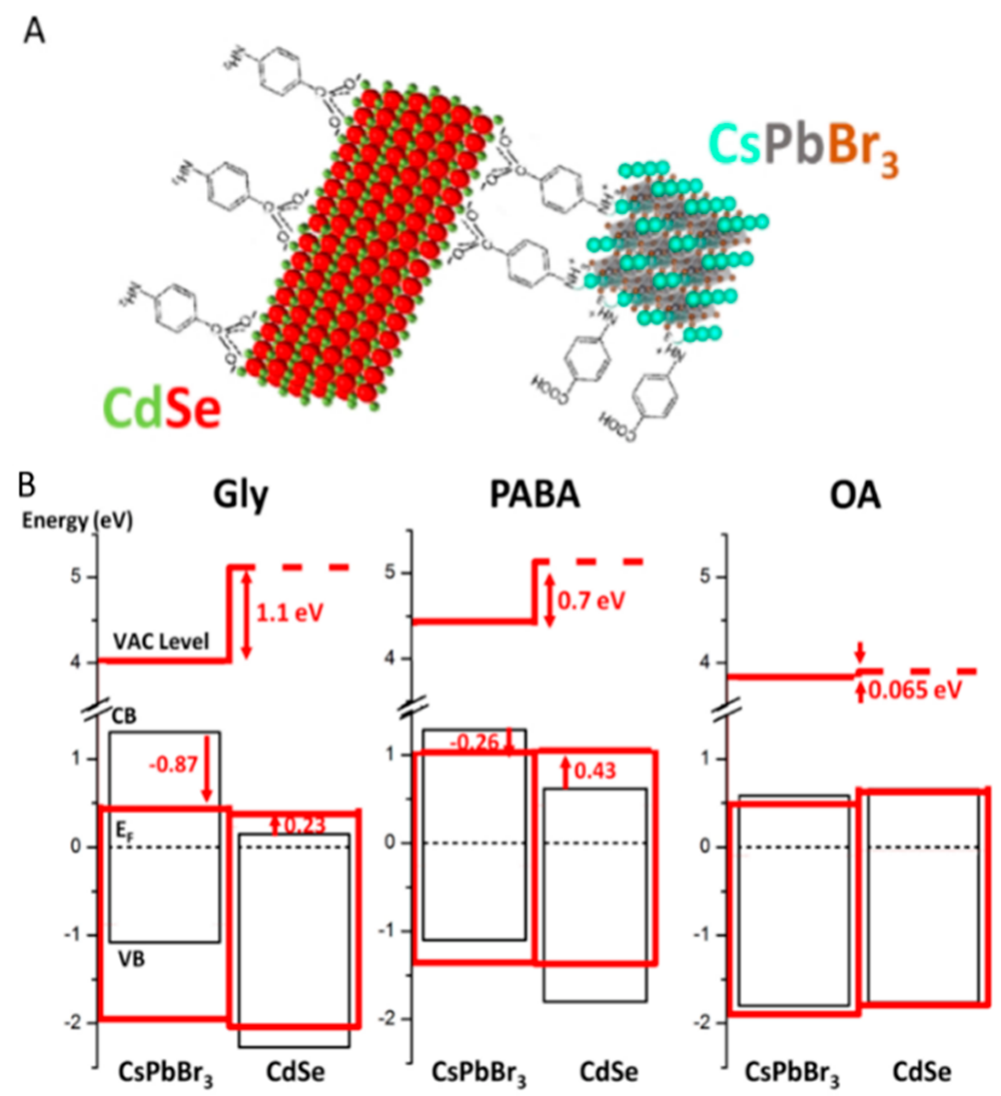
(A) Scheme depicting the CsPbBr_3_ NC linked by PABA to CdSe NPL. (B) Band diagrams of the hybrid systems (red lines) with indicated ligands, drawn relative to the system’s Fermi level. VB and CB of the pure systems are drawn as well (black lines), corresponding respectively to the top of the valence band and the bottom of the conduction band before complexation. Arrows indicate the electrostatic changes upon hybridization. The local vacuum level at the two constituents of the hybrid structure is indicated by the upper red lines. Technically, the vacuum level in the perovskite domains (indicted by the top red line) was extracted directly from the work-function measurements, whereas the one in the CdSe domains (top dashed red line) could not be resolved from secondary onsets and hence was deduced indirectly (with no compromise in accuracy) from the electrostatic information provided by the elemental core line shifts (an average over corresponding elements).

The surfaces of CsPbBr_3_ NPLs and CdSe NPLs with similar band gap energies were chemically modified to replace the native OA and OLA ligands with bifunctionalized ligands that have a carboxylic acid at one end and an amine at the other. Directional linking of the two materials at the single-particle level was achieved by preferential binding of the amine to CsPbBr_3_ and the carboxylic acid to CdSe and was verified by XPS elemental line analysis combined with FTIR measurements as depicted in the cartoon presented in Figure 5a. Comparing the binding energies of core-electron levels, as calculated from XPS measurements, of the hybrid CdSe/CsPbBr_3_ nanoparticle films with the corresponding ones made from the two individual nanomaterials (native capped CsPbBr_3_ and CdSe) gave information on the local electrostatic potential changes between the constituents upon complexation. Figure 5b illustrates the energy-level modifications upon hybridization. The red arrows in Figure 5b, pointing from black to red conduction bands (CBs), express the charge transfer, which is a consequence of Fermi level equilibration upon hybridization (depicted by the dashed black line). The effect is a step-like change in the local vacuum level that opens up, similar to a p–n junction, with increased electron density at the CdSe (which serves as the p-type component) and reduced electron density at the perovskite (the n-type analogue). Our results show significant electron transfer from the perovskite to the CdSe when linked by PABA and even more so by the Gly molecules with total (relative) changes of 700 and 1095 meV in binding energies, respectively. In contrast, with the electrically insulating long OA capping molecules this effect is very minor, well below 100 meV.

Owing to the nano dimensions of the hybrid system, the distribution of space–charge becomes an intriguing question. The preserved XPS elemental line widths suggest that the two constituent particles stabilize a roughly uniform potential, which suggests that the interface field is restricted to the molecular linkers. This can be justified by the fact that these molecules are inherently dipolar and therefore can withstand the electrostatic load. However, if the transferred charge is restricted to the interface, then one should encounter significant line broadening, which is not the case here, as verified experimentally. This observation is due to the limited (nano) size of the two constituents, which leads to space–charge that is distributed effectively over the entire particle. Hence, the interface field is believed to be predominantly restricted to the spacer, the linking molecular layer.

Complementary to the charge transfer at equilibrium, another photoinduced charge-transfer process was investigated by XPS analyses of the samples’ photoresponse to in situ white light illumination, thus gaining accessibility to the photodynamics in the hybrid system. A clear signature of electron transfer from the CdSe to the perovskite domains is revealed by opposite response signs at the two constituents. The photovoltage values found for the perovskite elemental lines in the PABA hybrid are, on average, 120 mV, whereas for CdSe we get 35 mV, reflecting a steady-state situation where the average charge density on each side of the junction is modified by the photoinduced electron transfer from the electron-rich CdSe side to the perovskite across the molecular layer. Accordingly, for the OA-capped hybrid, where the interface field is negligible, no photoinduced charge transport is observed. The light-induced XPS findings described above are consistent with measurements of the optical properties of hybrid films, such as absorption, PL, and PL lifetime measurements. Both the red-shifted PL and the extended PL lifetimes upon hybridization suggest radiative recombination originating from excitons that are spatially delocalized across the hybrid material, thus decreasing the overlap of electron and hole wave functions. The small extent of this effect is in line with the nearly zero band offsets deduced from XPS. Notably, the XPS complemented by in situ photovoltage measurements revealed that the type II emission properties have only a residual contribution over the pronounced charge-transfer effects driven by a large built-in field, restricted to the very narrow domain of the linking molecules at the CsPbBr_3_/CdSe NPL hybrids interface.

## 5 Summary and Perspectives

Although colloidal LHP NCs have an advantage over perovskite thin films in terms of production costs and material synthesis versatility, there are inherent challenges concerning their ligand passivation. Delicate changes in the ligand environment can have a dramatic effect on the stoichiometry, crystal structure, and habit of LHP NCs and consequently on device stability. Small changes in OA to OLAm Brønsted acid–base-type equilibrium promote, for example, the transition from cubic to orthorhombic CsPbI_3_ or the reversible transformation from cubic CsPbI_3_ to rhombohedral Cs_4_PbI_6_. Surface analysis suggests that phase transformations occur through a thermodynamic surface stabilization provided by the ligand shell. For Cs_4_PbBr_6_, the surface is passivated by an oleate–ammonium complex that can better stabilize the overall neutral charged surface composed of negatively charged PbBr_6_ octahedra partially balanced by in-plane Cs^+^ ions. In contrast, for CsPbBr_3_, the highly ionic nature of the surface requires a strong ionic stabilization where ammonium ligands partially substitute for Cs^+^ in the terminating A sites. Altogether, a rich pathway for controlling and stabilizing both the stoichiometry and structure of perovskite NCs in situ and postsynthesis was demonstrated.

Surface passivation also plays an important role at the interface between two materials, determining charge carrier generation and extraction efficiencies. A p–n-like junction between CsPbBr_3_ and CdSe NPLs, linked molecular ligands, was demonstrated. A pronounced charge-transfer effect driven by a large built-in field was demonstrated, controlled by the choice of ligand, changing the effective band diagram of the hybrid material. Interestingly, these large built-in fields are restricted to the very narrow domain of the linking molecules at the CsPbBr_3_/CdSe NPL hybrids interface. To conclude, insights gained from LHP NC surface analysis can aid in improving their stability, leading to their enhanced performance in applications such as photovoltaics and optoelectronics and possibly as a more sensitive test bed for the study of effects that are crucial to the long-term stability of LHP-based devices, such as self-healing.^52^ Finally, the use of surface ligands offers an interesting knob for generating and harnessing internal built-in fields in heterogeneous nanocrystal solids toward the implementation of these in new types of optoelectronic devices.
